# Core needle biopsy of breast tumours: comparison of diagnostic performance between surgery and radiology services at a national cancer centre in Latin America

**DOI:** 10.3332/ecancer.2024.1766

**Published:** 2024-09-13

**Authors:** Gonzalo Javier Ziegler-Rodriguez, Miguel Ángel Pinillos Portella, Gabriel De la Cruz Ku, Sheila Eunice Vílchez Santillan, Jorge Dunstan Yataco, José Antonio Galarreta Zegarra, Gabriela Calderón Valencia, José Manuel Cotrina Concha

**Affiliations:** 1 Department of Breast and Soft Tissue Tumor Surgery, National Cancer Institute of Peru (INEN), Lima 15038, Peru; 2 Senology Unit, Clinica Ziegler, Lima 15036, Peru; 3 School of Medicine, Universidad Peruana de Ciencias Aplicadas, Lima 15023, Peru; 4 Universidad Cientifica del Sur, Lima 15067, Peru

**Keywords:** core needle biopsy, ultrasound-guided biopsy of the breast, breast surgical oncologist, interventional radiologist, breast diagnosis

## Abstract

**Introduction:**

Breast pathology is a very common reason for medical attention. Tissue diagnosis is usually obtained with core needle biopsy which could be performed by breast surgeons or interventional radiologists. Our aim was to assess the comparison of diagnostic performance between the two services.

**Methods:**

A retrospective, descriptive and cross-sectional study was carried out on patients who had breast pathology at Instituto Nacional de Enfermedades Neoplasicas in 2019. Descriptive analyses, sensitivity and specificity were calculated using the R program version 4.2.3.

**Results:**

From 1,082 patients with breast tumours who underwent core needle biopsy (CNB) during 2019, 782 cases were included. Breast surgeons performed 462 CNBs and radiologists performed 320 CNBs. The 87.5% were palpable tumours and 525 breast carcinomas were identified in the final pathology. The diagnostic performance showed that the sensitivity and specificity were greater than 95% and 98%, respectively. The waiting time in both showed that >95% underwent a CNB before 2 months. The breast surgery service performed the majority of the biopsies in less than 1 week since the indication of the execution of the CNB compared to the radiology service (90% versus 36%).

**Conclusion:**

Both hospital services, breast surgery and radiology, are efficient in determining an accurate diagnosis using CNB. However, the breast surgery service performs CNB in a shorter time interval. Breast surgical oncologists are encouraged to perform CNB if there are understaffed radiology services to expedite the diagnosis and treatment of breast cancer patients.

## Introduction

Breast pathology is one of the most frequent reasons for medical attention around the world. Breast cancer is the second most common pathology of the breast, behind benign breast conditions [1]. In developed countries, public prevention policies are established with the strategy of screening with mammography for women between 50 and 69 years old. This, in addition to the well-organised health services, ensures that asymptomatic patients reach a radiology service dedicated to breast cancer to diagnose malignant lesions at an earlier stage, which allows improvement of disease-free and overall survival [2–4]. These tissue diagnoses are usually obtained with core needle biopsy (CNB) which can be performed by breast surgical oncologists or radiologists; however, in developed countries, the radiology service is usually responsible for these procedures with high accuracy and precision [5].

In developing countries like Peru, the estimated age-adjusted incidence of breast cancer is 35.9 per 100,000 inhabitants, which is not too high if we compare it to a developed country like Spain with 77.5 cases per 100,000 inhabitants [6]. However, unfortunately, Peru does not have efficient public policies for cancer prevention and, despite breast cancer being the second cancer with the highest incidence, a public mammographic screening programme practically does not exist [7]. The public system is subdivided according to the entity in charge of the finances such as social security, the ministry of health and other entities [8]. This fact creates high heterogeneity in the service, infrastructure, processes, referrals and in the academic level of professionals who serve. Moreover, there are gaps in access to health services, where the government has not been able to intervene efficiently, public hospitals do not typically have a dedicated breast surgery service and radiology services do not typically have a distinct breast imaging area [9].

All these limitations have a high impact on the outcomes of our population when compared to other developed countries, highlighting the importance of the early diagnosis of breast cancer to continue multidisciplinary treatment [10–13]. Hence, our aim was to compare the results of CNB obtained by the breast surgery service and the breast imaging radiology service in a tertiary reference hospital.

## Materials and methods

A retrospective, observational, descriptive and cross-sectional study was conducted. The research protocol was approved by the institutional review committee (22-0250/INEN). The data collected was from 12 months from 1st January 2019, to 31st December 2019, from the medical records of patients who underwent CNB in a breast surgery service (10 surgeons) and the radiology service (6 radiologists).

Our inclusion criteria were a) Patients with a breast tumour, b) undergoing CNB at the institute, c) performed by breast surgeons in the clinic or by radiologists from the breast imaging area and d) patients who had breast surgery with an operating piece following the CNB. Our exclusion criteria were a) CNB performed in the inpatient setting or emergency department and b) by other specialists inside or outside the institution.

### Variables

We included demographic, clinical and imaging characteristics, type of biopsies, number of attempts, waiting times and concordance with the final surgical specimen. We STARD guidelines were applied to all the biopsies as per protocol [14]. Patients were followed for at least 1 year since the indication of the CNB.

### Statistical analysis

We performed a descriptive analysis of the demographic and clinicopathological information, frequencies and percentages were used for qualitative variables, while mean with standard deviation or median with range for quantitative variables. Bivariate analyses were performed with chi-square for qualitative variables according to the service which performed by the CNB. Sensitivity and specificity were assessed for both services. A *p*-value <0.05 was considered statistically significant for all the analyses performed. The R-4.3.3 program was for data processing.

## Results

### Demographic characteristics

Of 1,082 cases, 782 patients were included in the present study. The 99.6% of cases were women with a median age of 51 years (range 14–92 years). Moreover, two thirds of the population (63.2%) resided in Lima with better accessibility to the hospital and 93.1% of patients did not have a personal history of breast cancer Table 1.

### Clinical presentation

Most of the cases presented with a palpable breast lesion (87.5%), and after a first breast surgery clinic appointment, 67.8% of patients had suspected breast cancer and were referred to different additional studies. Then, of these patients, 95.1% had a diagnostic ultrasound and 87.2% had a mammogram Table 1.

### CNB according to service and equipment used to guide the sample collection

The CNB was performed according to medical criteria, availability of staff and material in the pharmacy (14-gauge needle and compatible biopsy gun). Of the 782 patients who underwent CNB, 462 (59.07%) were performed by breast surgeons and 320 (40.92%) by breast radiology. Regarding the use of equipment to guide the sample collection, 452 (57.82%) clinical records did not specify the equipment used such as the ultrasound machine, most of them were in the breast surgery service. In 314 (40.2%) cases, ultrasound guidance was used to assist the CNB, while the rest (2.0%) was performed by radiology using stereotactic guidance Table 2.

### Need to repeat the biopsy and pathology results

Repeating the biopsy was not required in 714 (91.3%) cases; of them, 92.9% was performed by surgery and 89.1% by radiologist (*p* < 0.05). A total of 68 (8.7%) cases required a repeated biopsy. One group underwent surgery (surgical biopsy) (4.7%), and another group underwent re-BAG (4.0%). From the group with insufficient tissue who underwent CNB with the radiology department (35 patients), surgical consultation was obtained in 26 patients Table 2.

### Pathologic diagnosis

The pathology report obtained after the CNB identified 476 (60.9%) cases of infiltrating breast carcinomas and 29 (3.7%) cases of ductal carcinoma *in situ*, with a total of 505 of the 782 patients susceptible to undergo treatment for ‘breast cancer’ Table 3.

### CNB operational performance

To assess the operational performance of CNB by both executors, validity measures were calculated according to the success in obtaining sufficient samples to reach a tissue diagnosis. Sensitivity and specificity of both were similar for both groups; sensitivity of 95.3% and 95.6%, and specificity of 98.3% and 98.5%, for the breast surgical oncologist and radiologist, respectively Table 4.

### Evaluation of the waiting time to perform a CNB in both services

There was a difference between services regarding the waiting time, while breast surgeons performed 89.8% of cases during the first week after the procedure was indicated, radiologists were able to perform the CNB in 36.6% of their cases in this timeframe (*p* < 0.05). Specifically for patients who were diagnosed of ‘breast carcinomas’ (*n* = 525), no service took more than 2 months to perform a CNB, except for a minimal number of patients (<5%) Table 5.

## Discussion

In the context of a deficient health system, the breast surgical oncologist has served to cover gaps from other professionals such as the shortage of radiologists dedicated to breast cancer, in the diagnosis through a CNB. One of the main roles of the breast surgical oncologist is to evaluate the operability of the case, as well as to be the treating doctor of the patient with breast pathology [15]. Our study showed that both services have high sensitivity and specificity regardless of the tool used to perform the CNB; however, breast surgical oncologists can do it in a shorter period of time since the indication to the biopsy compared to radiologists.

In 2017, a total of 52 mammography machines were reported in Peru, of which only 37 were operational. In a country of 33 million inhabitants, it is almost one mammogram per 1,000,000 inhabitants [7]. In addition, mammographic screening is not performed in several regions, despite being encouraged by several institutions and national health programmes such as ‘Plan Esperanza’ [16]. Unfortunately, this is a reality that is not repeated in other countries, where services are better distributed and access to quality health is more equitable [12, 17]. In this setting, the surgical oncology service has adapted to help close the gap that there are not enough radiologists or advanced practice radiographers available to be able to care for patients seeking help due to breast tumours [18, 19].

In our study, the majority of cases were from patients older than 45 years with a median age of 55.9 years, similar to the cases of breast cancer reported in the Lima population registry and other studies [20, 21]. Around 88% of patients went to the hospital for a symptomatic breast, which was usually a palpable breast tumour, and was evaluated by the breast surgeon, being the professional they first had in a clinic appointment. Moreover, two thirds of cases had suspected cancer, and one third came from other places other than Lima, which represents poor access to oncological care due to socioeconomic inequalities [22, 23]. As mentioned, there are several barriers that contribute to a late diagnosis and worse outcomes in developing countries, such as an inappropriate reassurance of a questionable benign mass, the need for education and awareness, access to health care, inappropriate referrals, established mammography screening programmes, long waiting times for clinic appointments, among others. There is an urgent need for new health policies, strategies and cancer programmes throughout the country to achieve improvement in our oncological indicators, especially in underserved communities [24–26].

Since around two decades ago, the CNB has been used for the diagnosis of breast pathology and has served to de-escalate costs and morbidity in breast diagnosis, while the place of surgery to establish the diagnosis of a breast tumour has been discontinued [27, 28]. Furthermore, technology has improved significantly with the combination of images to guide the CNB, becoming a key part of the diagnosis [29]. For instance, the association of CNB with ultrasound guidance is the ideal combination similar to stereotactic biopsies if the mass is unable to be palpated or seen in ultrasound but able to be identified in mammography or digital tomosynthesis [30, 31].

Several surgical specialties, including breast surgeons, have adapted throughout the years to minimally invasive diagnosis [28]. Also, radiologists have entered the interventional field with equipment and techniques that allow small tumours to be removed, due to the benefit to the patient in terms of morbidity and costs. To date, CNB with or without ultrasound guidance is considered one of the quality standards in the diagnosis of breast cancer [30]. This is why within the competencies of the breast surgical oncologist, ultrasound training is crucial for the triage of lesions and guiding procedures such as fine needle aspiration, vacuum aspiration biopsy (VAB) and placement of marking devices [32–35]. It has been demonstrated that surgical oncologists have outstanding outcomes with this tool in the outpatient setting and intraoperatively for excisional biopsies or lymph node excision, for instance, it has shown reduced need for wire-guided localisation of impalpable tumours [36–38]. Our study showed that the results of both services were similar in sensitivity and specificity, highlighting that there is no difference in the accuracy between specialties.

In developed countries where the population screening programme and the radiology service can be dedicated to the breast, biopsies are usually performed by radiologists [39]. However in developing countries where services are not entirely well organised or radiologists are not available, breast surgical oncologists have continued to perform non-surgical biopsies, mastering ultrasound as a tool to guide the collection of samples with CNB, FNA or VAB [40]. Currently, there are several opportunities to be trained in the use of ultrasound for procedures and surgeries such as specialised courses endorsed by important scientific societies [32, 33, 35, 41]. These capabilities are not intended to replace the work of a radiologist, much less fall into intrusiveness, but rather, as in other medical specialties, the use of the ultrasound in the hands of the surgeon complements the assessment and helps with early diagnosis.

The high operational performance rates of the BAG in terms of concordance, sensitivity and specificity with or without ultrasound guidance in the present study demonstrate that the professionals of both services in our hospital are optimal options for the diagnosis of breast cancer [21, 42, 43]. Furthermore, the Kappa indices 0.92 show an optimal concordance between the pathology of the CNB and the final surgical pathology results in both groups, which were similar or higher than previous studies [44–46].

Furthermore, our study showed that the surgery department performed most of the biopsies within a week since the indication, while only a third of the patients underwent this procedure by the radiology department and approximately 45% had the biopsy in 2–3 weeks. This might be due to the dynamics of the treating physician and the low availability of radiologists to perform procedures on the first visit. The surgeon can perform the CNB at the same time as the first visit, while in radiology procedures are usually scheduled according to available appointments after 2–3 weeks. Moreover, the number of breast surgeons is greater than the number of radiologists who work on breast imaging. These delays in our study emphasizes the long waiting time caused by the hospital system, a consequence of the national health system [47]. Moreover, the an urgent need for more radiology specialists in tertiary centres, and even more in community hospitals. The reference is 2 months to consider the diagnostic study in cancer cases, with the aim to start oncological treatment before 90 days to avoid impact on survival [3, 27]. In our study, around 3% and 2% of the patients had the CNB out of this timeframe for radiologists and breast surgical oncologists, respectively, which should be improved to offer the best treatment and outcome to the patients.

Breast cancer requires a multidisciplinary approach to have the best outcome for the patient [48]. Our results encourage the breast surgeons to continue to perform biopsies with ultrasound guidance when needed, and the purpose is to work together with the radiologist to achieve an early diagnosis of the patient and start the treatment as earliest as possible when resources are scarce due to staffing, schedules, shifts and referrals [49, 50]. To date, breast cancer incidence continues to increase and all the possible resources should be used in the most effective and efficient way, especially in countries with gaps in the health system [10, 51].

Our study has some limitations. With two different services, a comparison is difficult due to the heterogeneity of cases and the clinical context of both services; however, we presented and evaluated the data according to the performance of both CNB executors in a tertiary referral centre. The size of the breast tumours subjected to biopsy was not known, the only information available was the label ‘palpable tumour’ and ‘non-palpable tumour’. This is because we have focused on the performance of CNB by two different specialists, hence we did not consider the specific characteristics of the breast tumours that underwent CNB such as histology, biology or stage. There was no category of patient-dependent or physician-dependent delays in the context of diagnostic delays. Delays in starting cancer treatment after a pathology diagnosis were also not collected. Furthermore, we have not specified how many surgeons or radiologists took the biopsies. We did not find records of the use of ultrasound equipment in the surgery department, but based on our experience we estimate that the surgeons used ultrasound guidance in a much higher percentage than found in the clinical history reports, then we highly recommend the use of this tool by breast surgeons based on previous literature. Moreover, our results should be interpreted with caution when extrapolation is intended, as our study only included one institution, hence, we recommend future multicentric prospective studies that compare both services for the diagnosis of breast cancer. In addition, despite these potential biases, the cross-sectional and comparative design is ideal when the purpose of research is to establish the performance, precision or accuracy of a diagnostic test under evaluation, in terms of sensitivity and specificity.

## Conclusion

To date, some developing countries still have patients with breast tumours that are self-diagnosed by palpation and not by mammography, a reality that should not be ignored. We demonstrate that CNB can be performed by either well-trained breast surgical oncologist or radiologist, both as effective resources to define pathology diagnosis of breast tumours, with the help of tools such as ultrasound to add precision and accuracy. Moreover, optimisation of time is crucial, and the surgery department is usually faster in terms of sampling the tissue in a shorter time since the indication. These results encourage the breast surgical oncologist to perform CNB when possible, to expedite diagnosis and treatment of the patients to achieve better oncological outcomes.

## Conflicts of interest

The authors declare that they have no conflicts of interest.

## Funding

This research has not received specific support from public sector agencies, the commercial sector or non-profit entities.

## Ethical considerations

This manuscript has followed the work centre's protocols for the publication of patient data and was approved for publication by the institution’s research ethics committee. Privacy has been respected, keeping the patient’s identification data confidential. The use of informed consent was not required.

## Author contributions

GJZR: Conceptualisation – Ideas; visualisation; writing – original draft; writing – review and editing

MAPP: Conceptualisation – Ideas; visualisation; writing – original draft; writing – review and editing

GDK: Conceptualisation – Ideas; visualisation; writing – original draft; writing – review and editing

SEVS: writing – review and editing

JDY: writing – review and editing

JAGZ: writing – review and editing

GCV: writing – review and editing

JMCC: visualisation; writing – original draft; writing – review and editing.

All authors participated in the conception, design of the work, writing and interpretation of the results, for the preparation of the manuscript.

## Figures and Tables

**Table 1.** Sociodemographic and clinical characteristics of patients who underwent CNB.

	*N* = 782	%
Sex		
Female	779	99.6
Male	3	0.4
Age – years, median (range)	51 (14–92)	
Age groups (years)		
0–14	1	0.1
15–29	38	4.9
30–44	193	24.7
45–59	310	39.6
60–74	187	23.9
75+	53	6.8
Residence		
Lima (accessible to INEN)	494	63.2
Other zones (not accessible to INEN)	288	36.8
Family history of breast cancer		
No	728	93.1
Yes	54	6.9
Clinical presentation		
Non palpable mass	98	12.5
Palpable mass	684	87.5
Diagnostic presumption per breast surgical oncologist (first appointment)		
Breast cancer suspicion	530	67.8
Benign breast tumour	231	29.5
Other breast tumour	21	2.7

**Table 2.** Characteristics of the CNB at the Instituto Nacional de Enfermedades Neoplasicas according to the service.

	Total *N* = 782	Breast surgical oncologist (Surgery department) *N* = 462 (59.1%)	Radiologist (Radiology department) *N* = 320 (40.9%)	*p*-value
Equipment used to obtain the sample				
No register (palpation included)	452 (57.8%)	449 (97.2%)	3 (0.9%)	
Ultrasound	314 (40.2%)	13 (2.8%)	301 (94.1%)	
Stereotactic biopsy	16 (2.0%)	0 (0.0%)	16 (5.0%)	<0.05
Second attempt				
No	714 (91.3%)	429 (92.9%)	285 (89.1%)	
Surgery (excisional or incisional)	37 (4.7%)	11 (2.4%)	26 (8.1%)	
CNB	31 (4.0%)	22 (4.8%)	9 (2.8%)	<0.05

**Table 3.** Pathological characteristics of patients who underwent CNB.

	*N* = 782
Final result from the breast specimen	
Breast invasive carcinoma (all the subtypes)	476 (60.9%)
Breast carcinoma in situ (todos los subtipos)	29 (3.7%)
Fibrocystic changes	74 (9.5%)
Mastitis or inflammatory process	65 (8.3%)
Fibroepithelial neoplasm	49 (6.3%)
Papillary neoplasm	27 (3.5%)
Fibroadenoma	24 (3.1%)
Phyllodes tumour (all the subtypes)	23 (2.9%)
Other malignant neoplasms	15 (1.9%)

**Table 4.** Concordance and validity of the CNB and the pathology result of the surgical specimen according to the service who performed the biopsy.

	Pathology result of the specimen					
	All patients (*n* = 782)		Patients who underwent CNB with a surgeon (*n* = 462)		Patient who underwent CNB with a radiologist (*n* = 320)	
Pathology result	Breast cancer	No breast cancer	Breast cancer	No breast cancer	Breast cancer	No breast cancer
Breast cancer	501 (64.1%)	4 (0.5%)	326 (70.6%)	2 (0.4%)	175 (54.7%)	2 (0.6%)
No breast cancer	24 (3.1%)	253 (32.4%)	16 (3.5%)	118 (25.5%)	8 (2.5%)	135 (42.2%)
Kappa index	0.92		0.90		0.94	
Sensitivity	95.4%		95.3%		95.6%	
Specificity	98.4%		98.3%		98.5%	

**Table 5.** Time from the indication to the CNB according to the service that performed the procedure.

	Total *N* = 782	Breast surgical oncologist (Surgery department) *N* = 462	Radiologist (Radiology department) *N* = 320	*p* value
Time from the indication to the CNB				
<1 week	532 (68.0%)	415 (89.8%)	117 (36.6%)	
2–3 weeks	180 (23.0%)	32 (6.9%)	148 (46.2%)	
3–7 weeks	48 (6.1%)	5 (1.1%)	43 (13.4%)	<0.05
>7 weeks	17 (2.2%)	8 (1.7%)	9 (2.8%)	
Lost to follow-up*	5 (0.6%)	2 (0.4%)	3 (0.9%)	

* Lost to follow up: category when the waiting time was more than 6 months since the indication for CNB
